# Cardiac magnetic resonance characteristics and prognostic associations of hypertension-mediated left ventricular hypertrophy

**DOI:** 10.1093/ehjimp/qyaf168

**Published:** 2026-01-13

**Authors:** Hafiz Naderi, Stefan van Duijvenboden, Julia Ramírez, Sucharita Chadalavada, Elisa Rauseo, Nay Aung, Steffen E Petersen, Patricia B Munroe

**Affiliations:** William Harvey Research Institute, Queen Mary University of London, Charterhouse Square, London EC1M 6BQ, UK; Barts Heart Centre, St Bartholomew’s Hospital, Barts Health NHS Trust, West Smithfield, London, EC1A 7BE, UK; William Harvey Research Institute, Queen Mary University of London, Charterhouse Square, London EC1M 6BQ, UK; Big Data Institute, La Ka Shing Centre for Health Information and Discovery, University of Oxford, OX3 7LF, UK; William Harvey Research Institute, Queen Mary University of London, Charterhouse Square, London EC1M 6BQ, UK; Aragon Institute of Engineering Research, University of Zaragoza, Zaragoza, Spain; Centro de Investigación Biomédica en Red-Biomateriales, Bioingeniería y Nanomedicina, Zaragoza, Spain; William Harvey Research Institute, Queen Mary University of London, Charterhouse Square, London EC1M 6BQ, UK; Barts Heart Centre, St Bartholomew’s Hospital, Barts Health NHS Trust, West Smithfield, London, EC1A 7BE, UK; William Harvey Research Institute, Queen Mary University of London, Charterhouse Square, London EC1M 6BQ, UK; Barts Heart Centre, St Bartholomew’s Hospital, Barts Health NHS Trust, West Smithfield, London, EC1A 7BE, UK; William Harvey Research Institute, Queen Mary University of London, Charterhouse Square, London EC1M 6BQ, UK; Barts Heart Centre, St Bartholomew’s Hospital, Barts Health NHS Trust, West Smithfield, London, EC1A 7BE, UK; William Harvey Research Institute, Queen Mary University of London, Charterhouse Square, London EC1M 6BQ, UK; Barts Heart Centre, St Bartholomew’s Hospital, Barts Health NHS Trust, West Smithfield, London, EC1A 7BE, UK; William Harvey Research Institute, Queen Mary University of London, Charterhouse Square, London EC1M 6BQ, UK

**Keywords:** cardiac magnetic resonance, hypertension, left ventricular hypertrophy, cardiovascular outcomes

## Abstract

**Aims:**

Hypertension-mediated left ventricular hypertrophy (LVH) phenotypes: normal left ventricle (LV), LV remodelling, eccentric and concentric LVH have been reported using cardiac magnetic resonance (CMR). Although previous smaller studies have explored associations of these phenotypes with select CMR metrics, large population-based longitudinal data comparing their clinical trajectories are lacking. This study aimed to evaluate CMR characteristics across hypertension-mediated LVH phenotypes and their associations with incident cardiovascular outcomes.

**Methods and results:**

In the UK Biobank imaging cohort, 24 463 hypertensives were categorized into LVH phenotypes using CMR. Logistic regression models explored the relationship between phenotypes, setting normal LV as the reference, and CMR parameters as exposures. Cox proportional hazard models evaluated associations with incident major adverse cardiovascular events (MACE) and separately heart failure over a median follow-up of 4.9 years. Among the participants, 23 206 had normal LV, 889 LV remodelling, 253 eccentric and 115 concentric LVH. Hypertensives with eccentric LVH had the most impaired LV function using ejection fraction and strain, and those with concentric LVH had the highest T1 values and maximal wall thickness. Hypertensives with eccentric LVH were associated with a 2.5 times higher rate of MACE (HR 2.5, CI: 1.7–3.8) and 9 times higher heart failure event rates (HR 9.0, CI: 5.7–14.2). Hypertensives with concentric LVH had 4.1 times higher heart failure events rates (HR 4.1, CI: 1.8–9.3), and no association with MACE.

**Conclusion:**

In this large population study, we found distinct differences in CMR characteristics between hypertension-mediated LVH phenotypes with eccentric and concentric LVH exhibiting the worst prognosis.

## Graphical Abstract

**Graphical Abstract:**
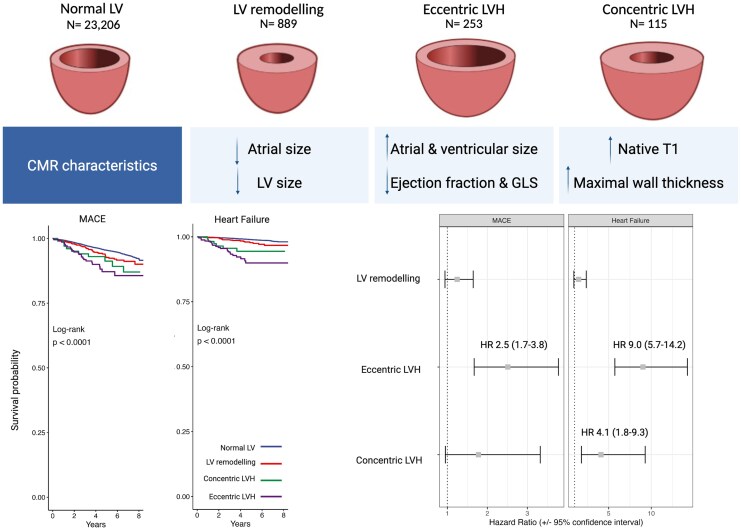

## Introduction

Hypertension is the most common cause of left ventricular hypertrophy (LVH), both strong predictors of cardiovascular morbidity and mortality.^1^ Importantly, LVH in the setting of hypertension is heterogeneous and can present with distinct left ventricular (LV) geometric patterns, ranging from normal geometry and LV remodelling to concentric and eccentric LVH, reflecting variation in chamber size defined using cardiac magnetic resonance (CMR) mass–to-volume relationships.^2,3^ In this study, we use the term hypertension-mediated LVH to describe this spectrum of myocardial adaptation in the context of blood pressure (BP) exposure after excluding alternative primary causes of LVH (e.g. inherited cardiomyopathy phenotypes).

Previous smaller studies have demonstrated that hypertension-mediated LVH phenotypes carry distinct myocardial signatures such as the differing burden of interstitial fibrosis, systolic myocardial strain and aortic distensibility using CMR when compared with normotensive individuals.^3–5^ These studies support biological plausibility for mass-to-volume based phenotype classification, however their modest sample size, selective recruitment and restricted outcome measures have limited generalizability. A large population-based longitudinal study comparing the clinical trajectories of these phenotypes is therefore lacking. An understanding of the CMR characteristics may in turn have treatment implications, with opportunities to tailor anti-hypertensive therapy for regression of LVH in hypertension.

In this study we explore the CMR structural and functional myocardial differences and clinical outcomes across hypertension-mediated LVH phenotypes using multiparametric data in a large community population. We hypothesized that CMR-defined eccentric and concentric LVH will correlate with impaired myocardial function and a greater burden of myocardial fibrosis, resulting in deleterious cardiovascular outcomes compared with normal LV geometry and LV remodelling phenotypes.

## Methods

### Study population

The UK Biobank (UKB) is a large prospective population study where demographics, medication history, electronic health records, biomarkers and genomics were collected in half a million participants aged 40–69 years when recruited between 2006 and 2010 from across the United Kingdom. The UKB imaging study was launched in 2015 with the aim of scanning 20% of the original cohort, that is 100 000 participants.^6^

**Figure 1:**
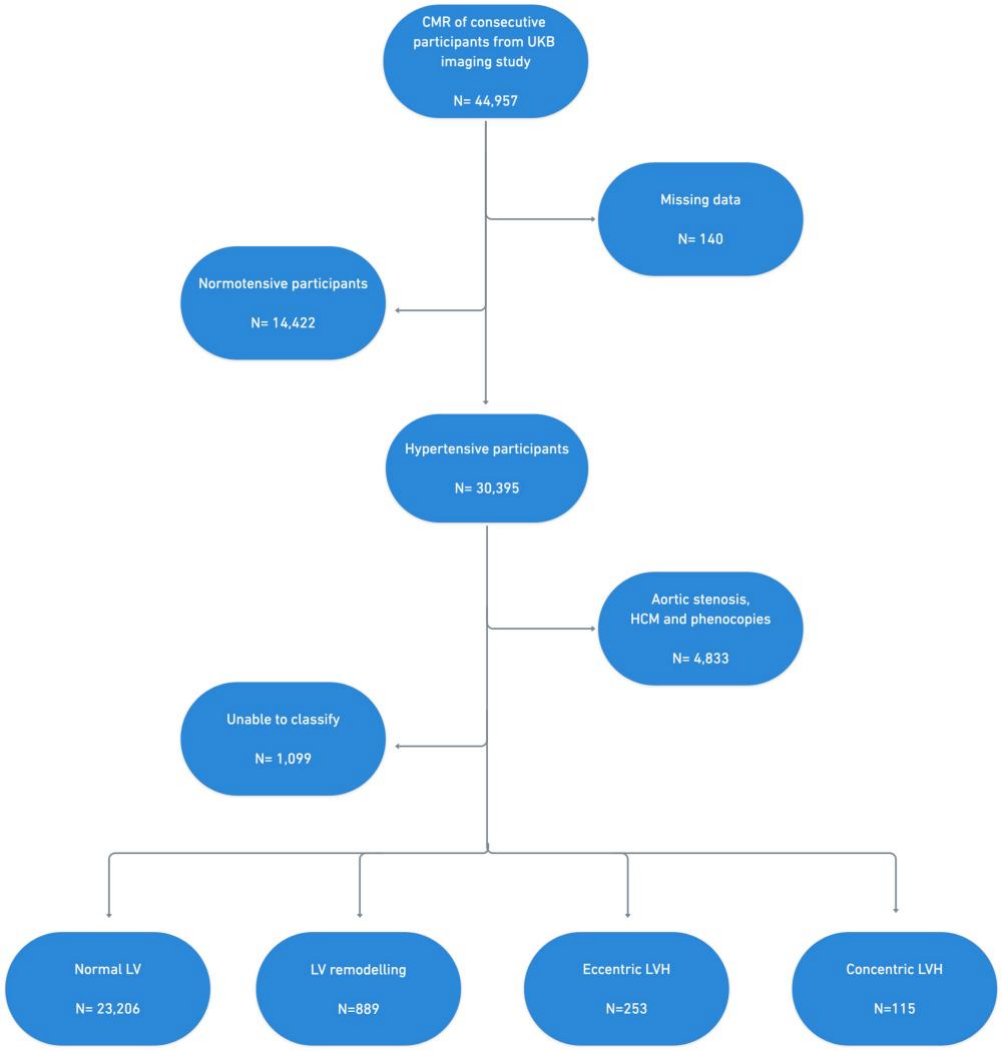
Flow diagram illustrating the categorization of the UK Biobank imaging cohort participants into the hypertension-mediated LVH phenotypes. CMR: Cardiac magnetic resonance, HCM, hypertrophic cardiomyopathy; LVH, left ventricular hypertrophy.

*Figure 1* illustrates the UKB sample selection process. A total of 44 957 participants had completed the UKB imaging study at the time of analysis. Hypertensive participants (*n* = 30 395) were identified according to the ‘high normal’ blood pressure (BP) grade of greater than or equal to 130/85 mmHg in the 2023 European Society of Hypertension (ESH) guideline.^7^ The 130/85 mmHg BP cut-off was used to identify subclinical disease as the risk of hypertension-mediated LVH is on a continuous exposure scale. BP measurements from the UKB imaging visit were analysed as these were taken on the same day as the CMR study. Each participant had two manual BP readings using a validated automated BP monitor or a manual sphygmomanometer. After calculating the average BP values, we adjusted for medication use by adding 15 and 10 mmHg to systolic BP and diastolic BP, respectively, for participants reported to be taking BP-lowering medication.^8,9^ We further defined hypertension by selecting relevant data fields from the UKB data showcase, including hypertension self-reported by participants, formal diagnosis from primary care physician and BP medication use (see Supplementary data online, *Table S1*). Participants with other causes of LVH (*n* = 4833) were excluded by reviewing exome sequence data for genes implicated in hypertrophic cardiomyopathy.^10^ The remaining hypertensive participants (*n* = 24 463) were categorized into the four hypertension-mediated LVH phenotypes using the mass-to-volume ratio. The CMR criteria used to define each phenotype are shown in *Table 1*. The LVH cut-offs were adapted using previously published work exploring CMR-based classification in hypertension.^3^ Indexing for body surface area was performed using the Mosteller formula.^11^

**Table 1:** Definition of left ventricular phenotypes by cardiac magnetic resonance parameters

	Normal LV		LV remodelling		Eccentric LVH		Concentric LVH	
	Male	Female	Male	Female	Male	Female	Male	Female
Index LV mass (g/m^2^)	≤70	≤55	≤70	≤55	>70	>55	>70	>55
Indexed end-diastolic volume (mL/m^2^)	≤110	≤94	≤110	≤94	>110	>94	≤110	≤94
LV mass: volume ratio (g/mL)	≤0.84	≤0.71	>0.84	>0.71	≤0.84	≤0.71	>0.84	>0.71

CMR, cardiac magnetic resonance imaging; LV, left ventricle; LVH, left ventricular hypertrophy. Indexing was performed using the Mosteller formula.

### CMR acquisition, image analysis and quality control

The CMR scans in UKB were performed using 1.5 Tesla scanners (MAGNETOM Aera, syngo Platform VD13A, Siemens Healthcare). The pre-specified UKB CMR protocol did not contain contrast administration and details of the CMR protocol have been described previously.^12^ The image analysis to derive volumetric data was performed using convolutional networks.^13^ The left atrial volumes were derived using Circle Cardiovascular Imaging, Inc. (cvi42 prototype 5.11.0). Global native T1 was included in the analysis, as a measure of myocardial tissue character. Myocardial T1 mapping was acquired in one mid-ventricular SAX slice using the Shortened Modified Look-Locker Inversion recovery technique (ShMOLLI) mapping sequence.^14^ Global myocardial native T1 was calculated using the fully automated software (cvi42 prototype 5.14.1.2875).^15^ The CMR-FT strain analysis was generated using automated batch processing (cvi42 prototype 5.13.7).^16^ Quality control of the data output was performed using the removal of statistical outliers and visual quality control described in a dedicated publication.^17^

### Ascertainment of cardiovascular outcomes

Longitudinal data on clinical outcomes of UKB participants is recorded using linkage to Hospital Episode Statistics (HES) and UK death register.^18^ The primary endpoint was major adverse cardiovascular events (MACE), defined as either hospitalization or death due to fatal/non-fatal myocardial infarction, stroke or ventricular arrhythmias. Cases were identified using relevant International Classification of Disease, 9th or 10th Revision (ICD-9, ICD-10), or Office of Population Censuses and Surveys version 4 (OPCS 4) Classification of Interventions and Procedures codes in the health-related records or death register (see Supplementary data online, *Table S2*). An additional analysis was performed testing for associations with heart failure. The follow-up period was determined by the first appearance of ICD-9, ICD-10 or OPSC4 codes in either health record or death register data since the UKB imaging visit. Participants with prevalent events at the time of UKB enrolment were excluded from the survival analyses. Participants who did not experience an event were censored at death or the end of the follow-up period (30 November 2022).

### Statistical analyses

Descriptive statistics are presented as median (interquartile range) for continuous variables or frequency (percentage) for categorical variables. The distribution of continuous data was assessed by visual inspection of the histograms and normality was evaluated using the Shapiro-Wilk test. Baseline clinical and CMR characteristics of the hypertension-mediated LVH phenotypes were statistically compared with the normal LV group. To assess for associations, the analysis of variance (ANOVA) test was used for continuous data and the chi-square test for categorical data. Following overall group comparisons using one-way analysis of variance (ANOVA), *post hoc* pairwise comparisons were performed using Tukey’s honestly significant difference (HSD) test for selected key CMR parameters. For all analyses, a two-tailed *P*-value <0.05 was deemed statistically significant. Statistical analyses were performed using R version 4.0.3 and RStudio Version 1.3.1093.^19^

After standardizing CMR values, the parameters with less than 10% of missing data were imputed using the Multivariate Imputation by Chained Equations (MICE) package, with five imputed datasets and ten iterations.^20^ For each variable, we specified a predictive mean matching model, and we used relevant variables to inform the imputation. Imputations were found acceptable by comparison of plots of the distribution of recorded and imputed values for all measurements. Logistic regression models were built to determine the relationship between each hypertension-mediated LVH phenotype as a binary outcome, setting normal LV as the reference group (e.g. concentric LVH vs. normal LV) and CMR parameters as exposure. CMR parameters that were used to define each LVH phenotype (e.g. LV mass, LVEDV, LV mass-to-volume ratio) were not included as exposures in the regression models to avoid potential collinearity. Classification And Regression Training (CARET) package was used for correlation analysis and highly correlated CMR parameters were also omitted (correlation coefficient threshold of +/– 0.7) to avoid collinearity. The mean absolute correlation of each CMR parameter was calculated, and the metrics with the largest mean absolute correlation were omitted (see Supplementary data online, *Figure S1*). For each LVH phenotype, two multivariable logistic regression models were built, unadjusted and clinically adjusted (for age, sex, body mass index and systolic BP). Logistic regression models were estimated separately for each imputed dataset and then averaged to obtain one overall set of estimates. To allow comparison of the magnitude of effects across CMR measures, odds ratios (OR) were reported with 95% confidence intervals (CI).

For testing LVH phenotypes with cardiovascular outcomes, Kaplan–Meier curves were constructed to show the survival probability over the follow-up period in each of the four hypertension-mediated LVH phenotypes for MACE and heart failure. The log-rank test was used to compare survival between groups. The association between hypertension-mediated LVH phenotypes and outcomes was tested using multivariable-adjusted Cox proportional hazard regression, adjusted for age, sex, BMI, systolic BP, diabetes, high cholesterol, and smoking status, setting normal LV as the reference group. In sensitivity analysis, associations were re-examined without adjustment of BP values of participants on anti-hypertensive medication. Hazard ratios (HR) were reported with 95% CI to derive risk for each LVH phenotype compared with the normal LV group.

## Results

### Baseline characteristics of participants

A total of 24 463 hypertensive participants of median age 66 years, 45.4% females, were included in this study. *Table 2* summarizes the baseline characteristics of the hypertensive participants in the UKB imaging study for this analysis, stratified by LVH phenotypes. Most hypertensive participants had normal LV (*n* = 23 206) on CMR imaging, followed by LV remodelling (*n* = 889), eccentric LVH (*n* = 253) and concentric LVH (*n* = 115). Among the hypertensive participants, the median BP was 149/85 mmHg with 36.8% of participants on anti-hypertensive therapy. Broadly, participants in the eccentric and concentric LVH groups had the highest average BP measurements. The proportion of males was higher (58.7%) in the eccentric LVH group, whereas more females (59.1%) had concentric LVH. The proportion of participants with high cholesterol (76.3%) and diabetes (15.2%) was highest in hypertensive participants with LV remodelling.

**Table 2:** Baseline characteristics of hypertensive participants in the UK Biobank imaging study

	Overall (*n* = 24 463)	Normal LV (*n* = 23 206)	LV remodelling (*n* = 889)	Eccentric LVH (*n* = 253)	Concentric LVH (*n* = 115)	*P*-value
Age (years)	66 [11]	66 [11]	68 [10]	65 [12]	68 [9]	<0.001
Sex (%)						<0.001
Female	11 117 (45.4)	10 414 (44.9)	528 (59.4)	107 (42.3)	68 (59.1)	
BMI (kg/m^2^)	26.7 [5.2]	26.6 [5.1]	28.4 [5.7]	25.8 [5.3]	27.3 [6.2]	<0.001
Ethnicity (%)						0.12
White European	23 847 (97.5)	22 636 (97.5)	856 (96.3)	244 (96.4)	111 (96.5)	
Other	494 (2.0)	457 (2.0)	25 (2.8)	8 (3.2)	<5 (3.5)	
Systolic BP (mmHg)	149 [24]	148 [23]	156 [26]	157 [28]	160 [32]	<0.001
Diastolic BP (mmHg)	85 [14]	85 [14]	88 [15]	85 [17]	89 [17]	<0.001
High cholesterol (%)	17 333 (70.9)	16 402 (70.7)	678 (76.3)	168 (66.4)	85 (73.9)	<0.001
Diabetes (%)	1710 (7.0)	1540 (6.6)	135 (15.2)	19 (7.5)	16 (13.9)	<0.001
Medication use						
BP-lowering medication (%)	9008 (36.8)	8401 (36.2)	437 (49.2)	106 (41.9)	64 (55.7)	<0.001
Lipid-lowering medication (%)	8008 (32.7)	7498 (32.3)	393 (44.2)	73 (28.9)	44 (38.3)	<0.001
Smoking status (%)						<0.001
Never	14 353 (58.7)	13 693 (59.0)	460 (51.7)	140 (55.3)	60 (52.2)	
Previous	8553 (35.0	8084 (34.8)	337 (37.9)	89 (35.1)	43 (37.4)	
Current	1435 (5.9)	1316 (5.7)	84 (9.4)	23 (9.1)	12 (10.4)	
Alcohol intake (%)						0.23
Never	1090 (4.5)	1022 (4.4)	52 (5.8)	11 (4.3)	5 (4.3)	
Current	23 251 (95.0)	22 071 (95.1)	829 (93.3)	241 (95.3)	110 (95.6)	

Counts variables are presented as number (percentage), continuous variables as median [interquartile range]. To assess for associations between LVH phenotypes, the analysis of variance (ANOVA) test was used for continuous data and χ^2^ test for categorical data. Blood pressure values are adjusted for medication use.

BMI, body mass index; BP, blood pressure; LV, left ventricle; LVH, left ventricular hypertrophy; mmHg, millimetres mercury.

### Association between CMR parameters and hypertension-mediated LVH phenotypes

*Table 3* shows the association between hypertension-mediated LVH phenotypes and CMR parameters. Overall, atrial and ventricular size were smallest in the LV remodelling phenotype and largest in participants with eccentric LVH. The highest T1 values and maximal wall thickness measurements were observed in hypertensive participants with concentric LVH and the lowest values in those with a normal LV pattern. In terms of functional measures, eccentric LVH had the most impaired LV and RV function measured using ejection fraction and myocardial strain. Functional CMR parameters were comparable among the other phenotypes. Tukey *post hoc* analysis demonstrated clear and consistent between-group differences across key CMR parameters (see Supplementary data online, *Table S3*). Eccentric LVH differed significantly from normal LV geometry for measures of systolic function, including GLS and LVEF, and for chamber size indices, indicating more advanced functional impairment. Concentric LVH showed the largest differences for global wall thickness and native T1 compared with other phenotypes, consistent with greater myocardial hypertrophy and fibrosis burden. Differences between LV remodelling and normal LV were generally smaller and less consistently significant.

**Table 3:** CMR myocardial structural and functional parameters of hypertensive participants in the UK Biobank imaging study

	Overall (*n* = 24 463)	Normal LV (*n* = 23 206)	LV remodelling (*n* = 889)	Eccentric LVH (*n* = 253)	Concentric LVH (*n* = 115)	*P*-value
Left ventricular parameters						
LV end-diastolic volume (LVEDV), ml	144 [44]	145 [43]	112 [29]	229 [89]	148 [49]	<0.001
Indexed LVEDV, mL/m^2^	76 [16]	76 [17]	58 [12]	109 [13]	79 [10]	<0.001
LV end-systolic volume (LVESV), ml	57 [24]	58 [24]	43 [16]	104 [46]	57 [24]	<0.001
Indexed LVESV, mL/m^2^	30 [10]	30 [10]	22 [7]	47 [7]	30 [11]	<0.001
LV stroke volume (LVSV), ml	86 [25]	86 [25]	68 [19]	115 [38]	89 [27]	<0.001
Indexed LVSV, mL/m^2^	45 [10]	45 [11]	35 [7]	58 [15]	46 [8]	<0.001
LV mass (LVM), g	87 [32]	87 [32]	89 [31]	135 [51]	115 [61]	<0.001
Indexed LVM, g/m^2^	45 [11]	45 [11]	46 [10]	64 [13]	58 [7]	<0.001
LV mass: volume (LVM/LVED), g/mL	0.6 [0.1]	0.6 [0.1]	0.8 [0.1]	0.6 [0.2]	0.9 [0.2]	<0.001
Indexed LVM/LVEDV, g/mL/m^2^	0.3 [0.1]	0.3 [0.1]	0.4 [0.1]	0.3 [0.1]	0.4 [0.1]	<0.001
Maximal LV wall thickness, mm	10 [2]	10 [2]	11 [2]	11 [2]	13 [3]	<0.001
LV ejection fraction, %	60 [8]	60 [8]	62 [8]	53 [13]	62 [9]	<0.001
Native T1 mapping, ms	927 [42]	927 [42]	931 [39]	933 [56]	947 [50]	<0.001
Global circumferential strain, %	−19 [8]	−19 [3]	−19 [3]	−17 [3]	−18 [3]	<0.001
Global radial strain, %	31 [8]	31 [8]	32 [7]	27 [7]	29 [9]	<0.001
Global longitudinal strain, %	−18 [7]	−18 [3]	−17 [3]	−17 [3]	−16 [3]	<0.001
Right ventricular parameters						
RV end-diastolic volume (RVEDV), mL	153 [51]	154 [51]	122 [36]	199 [68]	147 [49]	<0.001
Indexed RVEDV, mL/m^2^	80 [19]	80 [19]	63 [14]	101 [23]	79 [16]	<0.001
RV end-systolic volume (RVESV), ml	65 [29]	65 [29]	53 [21]	90 [37]	60 [26]	<0.001
Indexed RVESV, mL/m^2^	34 [2]	34 [12]	27 [9]	46 [3]	32 [11]	<0.001
RV stroke volume (RVSV), mL	88 [27]	88 [27]	70 [20]	108 [39]	88 [25]	<0.001
Indexed RVSV, mL/m^2^	46 [11]	46 [11]	36 [10]	55 [15]	46 [9]	<0.001
RV ejection fraction, %	58 [8]	58 [8]	57 [9]	54 [8]	58 [9]	<0.001
Atrial parameters						
Maximal left atrial (LA) volume, mL	40 [17]	40 [17]	33 [12]	57 [25]	46 [18]	<0.001
Indexed LA, mL/m^2^	21 [8]	21 [8]	17 [8]	28 [10]	23 [9]	<0.001
Maximal right atrial (RA) volume, mL	44 [21]	44 [21]	34 [15]	58 [27]	41 [18]	<0.001
Indexed RA, mL/m^2^	21 [11]	23 [11]	18 [8]	30 [12]	22 [11]	<0.001

Continuous variables presented as median [interquartile range]. To assess for associations between LVH phenotypes, the analysis of variance (ANOVA) test was used for continuous data.

g, grams; LV, left ventricle; LVH, left ventricular hypertrophy; mm, millimetre; mL, millilitre; ms, millisecond; m^2^, metres squared; RV, right ventricle.

**Figure 2:**
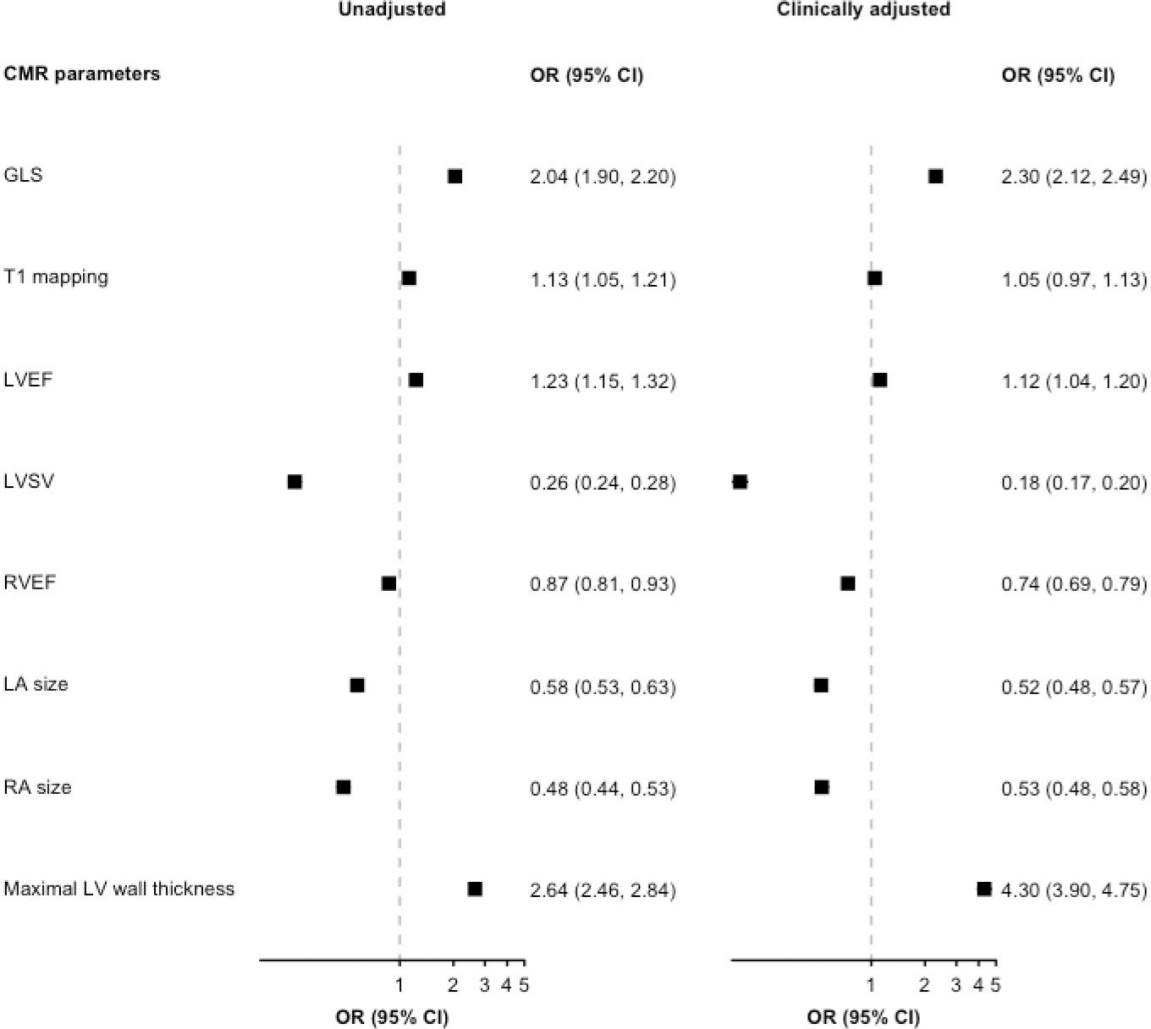
Association between CMR parameters and hypertension-mediated LV remodelling in unadjusted and clinically adjusted regression models. GLS, Global longitudinal strain; LVEF, left ventricular ejection fraction; LVSV, left ventricular stroke volume; RVEF, right ventricular ejection fraction; LA, left atrium; RA, right atrium; OR, Odds ratio; CI, confidence interval.

The logistic regression models showing the association between CMR parameters (exposure), and hypertension-mediated LV remodelling (outcome) are shown in *Figure 2*. With normal LV set as the reference group, there were significant negative associations between cardiac chamber volumes and LV remodelling, which persisted in the clinically adjusted models. For example, hypertensives with one standardized increase in LA and RA size were associated with 0.5 (OR 0.5, 95% CI: 0.5–0.6) and 0.5 (OR 0.5, 95% CI: 0.5–0.6) odds of LV remodelling, respectively. The hypertensive individuals with one standardized impairment in GLS had 2.3 times increased odds of LV remodelling (OR 2.3, 95% CI: 2.3–2.5), compared with a normal LV.

**Figure 3:**
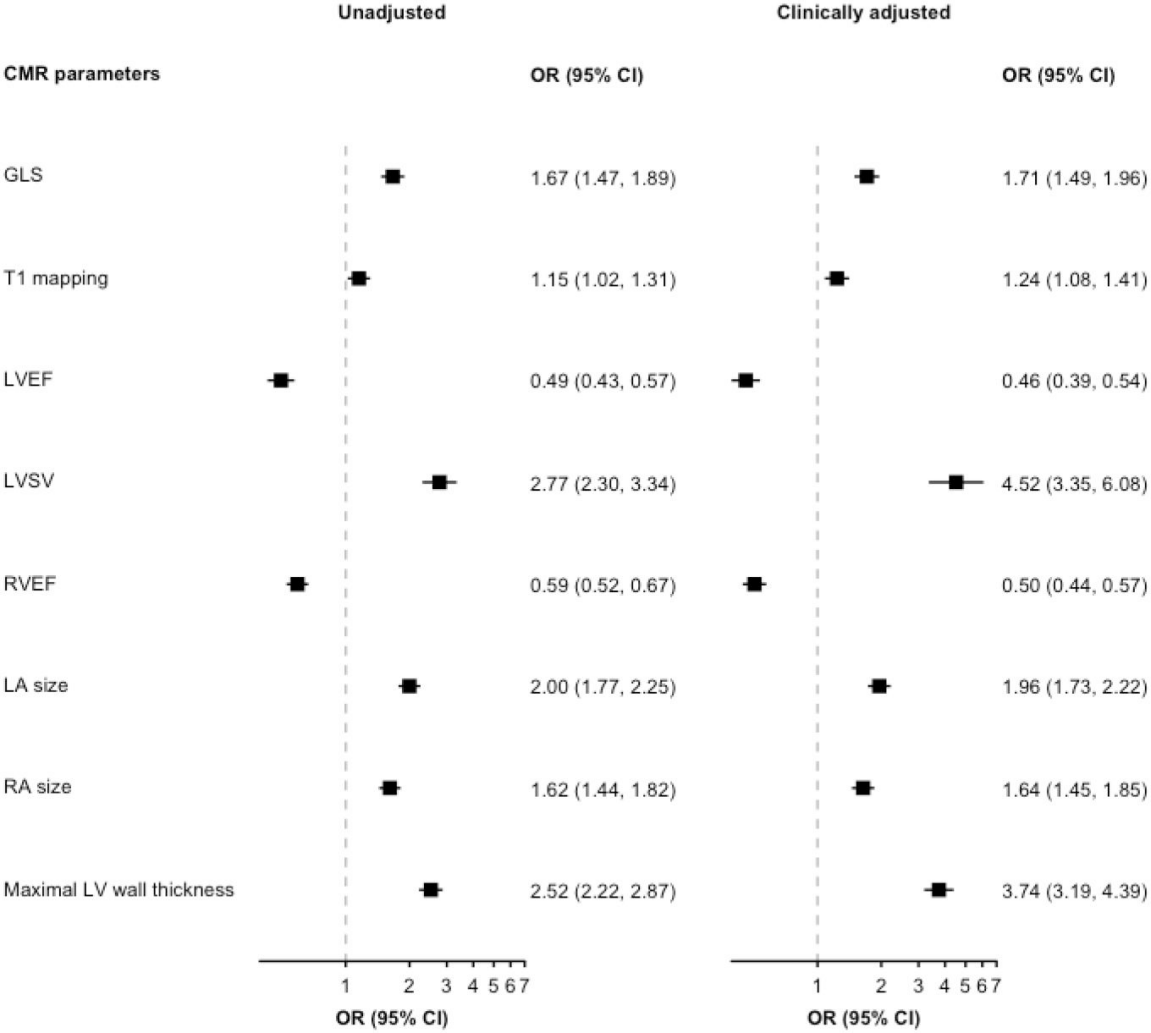
Association between CMR parameters and hypertension-mediated eccentric hypertrophy in unadjusted and clinically adjusted regression models. GLS, Global longitudinal strain; LVEF, left ventricular ejection fraction; LVSV, left ventricular stroke volume; RVEF, right ventricular ejection fraction; LA, left atrium; RA, right atrium; OR, Odds ratio; CI, confidence interval.

In contrast to LV remodelling, there were significant positive associations between cardiac chamber volumes and eccentric LVH, which remained significant in the clinically adjusted model (*Figure 3*). One standardized increase in LA and RA size had 1.9 times (OR 1.9, 95% CI: 1.7–2.2) and 1.6 times (1.6, 95% CI: 1.5–1.9) increased odds of eccentric LVH, respectively. The results also showed that hypertensives with worse LV function had increased odds of eccentric hypertrophy. For a standardized increase in LVEF, there was 0.5 odds (0.5, 95% CI: 0.4–0.5) of eccentric LVH in the clinically adjusted model.

**Figure 4:**
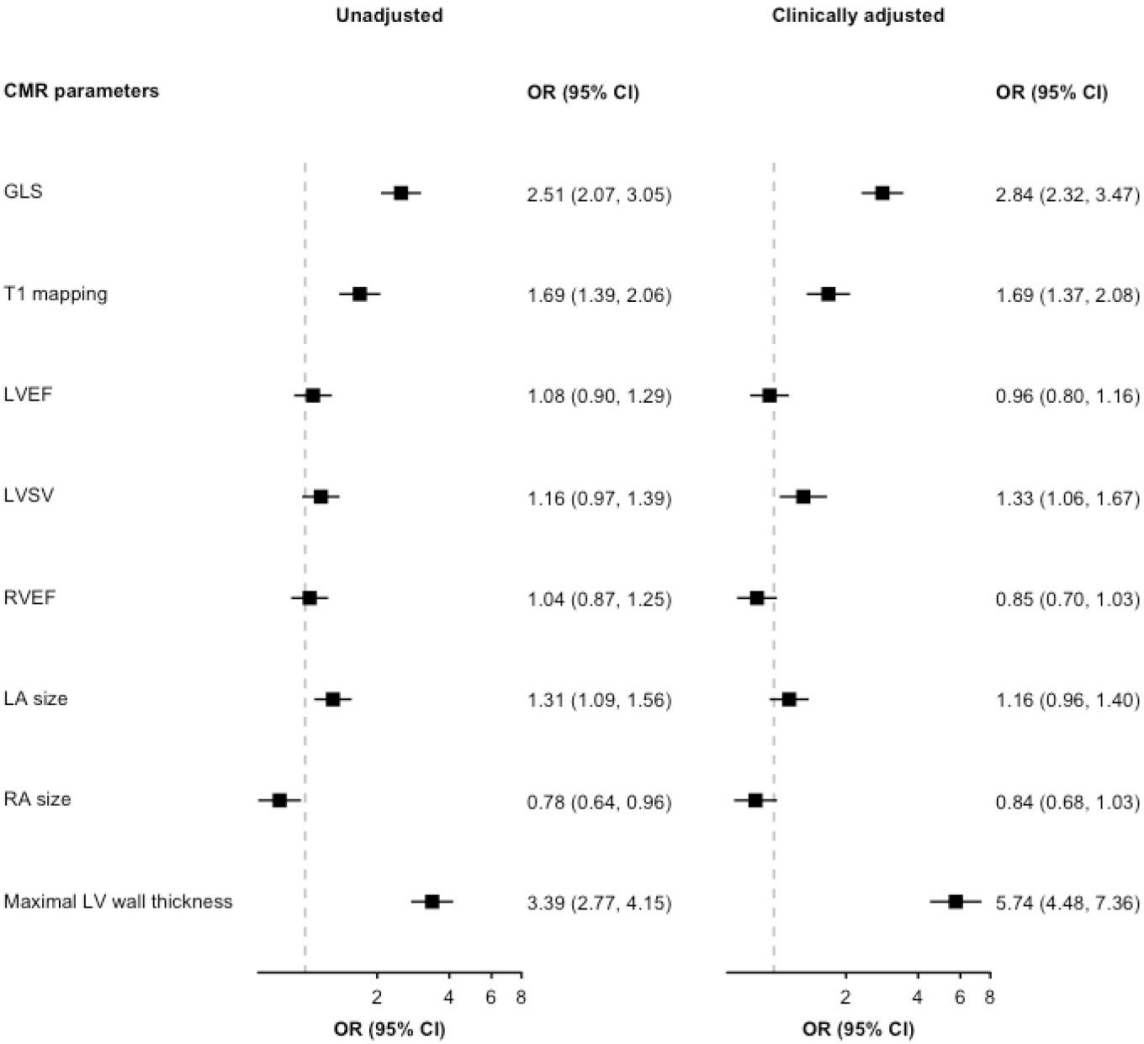
Association between CMR parameters and hypertension-mediated concentric hypertrophy in unadjusted and clinically adjusted regression models. GLS, Global longitudinal strain; LVEF, left ventricular ejection fraction; LVSV, left ventricular stroke volume; RVEF, right ventricular ejection fraction; LA, left atrium; RA, right atrium; OR, Odds ratio; CI, confidence interval.

The logistic regression models showing the association between CMR parameters and hypertension-mediated concentric LVH are shown in *Figure 4*. Compared with the normal LV as the reference group, the strongest association with concentric hypertrophy was seen with maximal wall thickness. In the clinically adjusted model, the odds of having concentric LVH were approximately 6 times greater (OR 5.7, 95% CI: 4.5–7.4) for every standardized increase in wall thickness. In contrast to LV remodelling and eccentric LVH groups, the associations with cardiac volumes were not statistically significant. In terms of myocardial strain, hypertensive participants with impaired GLS had approximately 3 times (OR 2.8, 95% CI: 2.3–3.5) increased odds of having concentric LVH in our clinically adjusted model. Furthermore, for every standardized increase in T1 measure, there was 1.7 times increased odds (OR 1.7, 95% CI: 1.4–2.1) of concentric LVH.

### Associations of hypertension-mediated LVH phenotypes with cardiovascular outcomes

**Figure 5:**
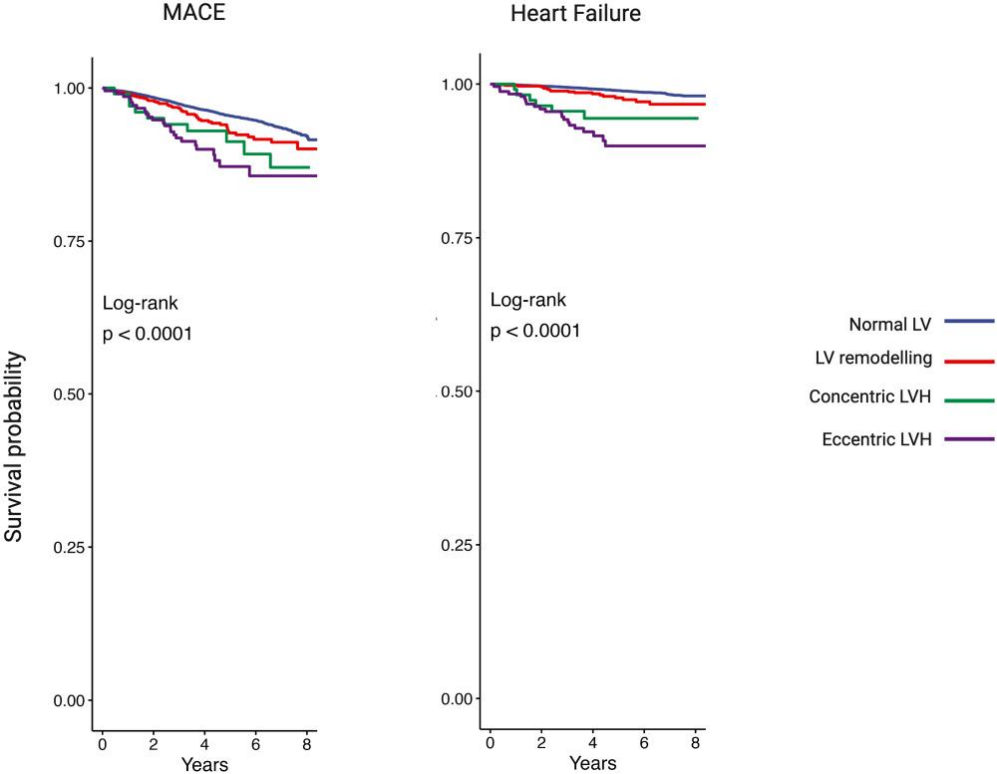
Kaplan–Meier curves showing the survival probability over the follow-up period. In each of the four hypertension-mediated LVH phenotypes for MACE and heart failure. LV, left ventricle, LVH, left ventricular hypertrophy, MACE, major adverse cardiovascular events.

Over a median follow-up period of 4.9 years the incident MACE and heart failure events were 1032 (4.2%) and 281 (1.1%), respectively. *Figure 5* shows differences in cumulative survival probability between hypertension-mediated LVH phenotypes, with a graphical representation of clinical outcomes occurrence. There was a statistically significant difference in survival from MACE (*P* < 0.001) and heart failure (*P* < 0.001) between the hypertensive-mediated LVH phenotypes using the log-rank test.

**Figure 6:**
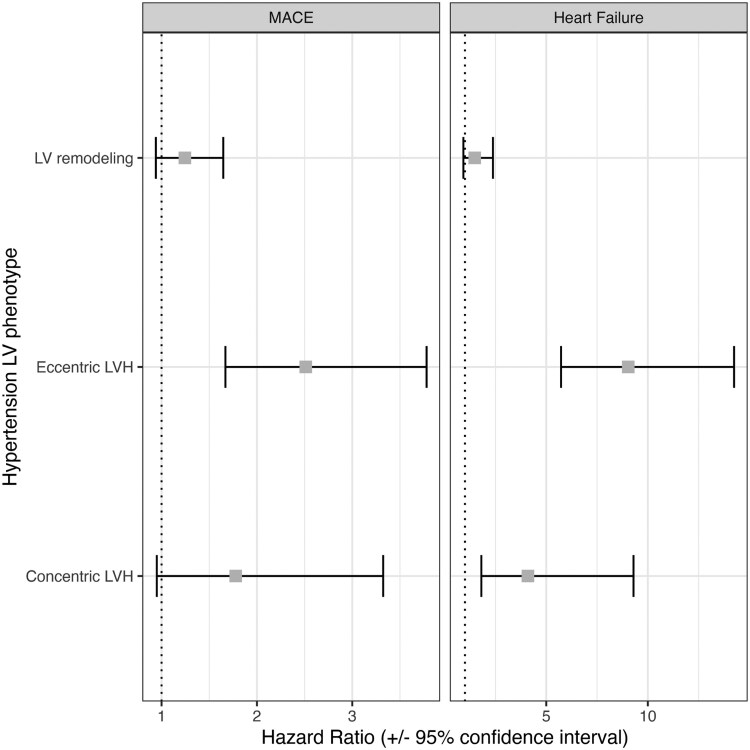
Associations of hypertension-mediated LVH phenotypes and clinical outcomes. Results are HRs from Cox hazards proportional regression models. The diseases listed are set as the model outcome (response variable) and hypertension-mediated LV phenotype in the exposure of interest with normal LV as the reference group. The model was adjusted for age, sex, BMI, and systolic BP, diabetes, high cholesterol and smoking status; MACE, major adverse cardiovascular event; LV, left ventricle; LVH, left ventricular hypertrophy.

*Figure 6* and Supplementary data online, *Table S4* show the hazard ratios using Cox regression, setting normal LV as the reference. The hazard rate of MACE was 2.5 times higher (HR 2.5, CI: 1.7–3.8) and heart failure 9 times higher (HR 9.0, CI: 5.7–14.2) in hypertensives with eccentric LVH. Hypertensives with concentric LVH were associated with 4.1 times higher heart failure event rates (HR 4.1, CI: 1.8–9.3) compared with normal LV, and there was no association with MACE. In sensitivity analysis, re-estimating the models without adjusting BP for anti-hypertensive therapy did not alter the direction or magnitude of associations, as shown in Supplementary data online, *Table S5*. Notably, some associations reached statistical significance in the absence of BP adjustment.

## Discussion

### Summary of findings

In this large population-based cohort of 24 463 hypertensives from UKB, most participants had a normal LV pattern (*n* = 23 206), with the smallest group having concentric LVH (*n* = 115), defined using CMR. Hypertensives in the eccentric and concentric LVH groups had the highest average BP measurements, despite having the greatest number of participants on anti-hypertensive medical therapy. There were distinct differences in CMR characteristics between hypertension-mediated LVH phenotypes and significant associations of eccentric and concentric LVH with incident cardiovascular outcomes.

### Associations with CMR parameters

To our knowledge, this is the largest study comparing hypertension-mediated LVH phenotypes using multiparametric CMR measures. Higher T1 values, thicker LV wall and worse GLS are associated with increased odds of LV remodelling, eccentric LVH and concentric LVH, compared with normal LV geometry. This may suggest that these phenotypes are among a spectrum, with LV remodelling pattern being an intermediary phenotype progressing to either concentric LVH or eccentric LVH when BP is not well controlled. Post hoc pairwise analyses further support the presence of distinct hypertension-mediated LVH phenotypes rather than a uniform hypertrophic response. The pronounced functional differences observed in eccentric LVH, particularly in measures of systolic function and chamber enlargement, are consistent with a more advanced maladaptive remodelling process. In contrast, concentric LVH was characterised by greater wall thickness and higher native T1 values, suggesting a predominantly pressure-overload driven phenotype with increased myocardial hypertrophy and fibrosis. The relatively modest and less consistent differences between LV remodelling and normal LV geometry are compatible with an intermediate or adaptive stage along the continuum of hypertensive cardiac remodelling.

Elghazaly *et al*.^21^ have also described hypertension-related CMR phenotypes in UKB, considering variations between hypertensive and normotensive individuals, with particular emphasis on sex and ethnic differences. In their study, associations between hypertension and CMR metrics were estimated using linear regression and the threshold for diagnosed hypertension was set at a systolic BP of 140 mmHg.^22^ Elghazaly *et al.* found that the prominent myocardial phenotype in UKB participants with hypertension was concentric LVH with lower myocardial native T1 and poorer LV function by GLS. In contrast, our results showed hypertensives with concentric LVH had the highest interstitial fibrosis demonstrated by raised T1 values, whereas hypertensives with eccentric LVH had the worst LV function. These divergent observations may arise from differences in study design and analytic approach. Elghazaly and colleagues defined concentric LVH solely based on LV mass and wall thickness, without stratification by geometric pattern or exclusion of alternative causes of LVH using genetic data. While the prevalence of inherited cardiomyopathies in UK Biobank is low, their inclusion in a large population sample (*n*∼5000) could still exert a downward influence on average native T1 values. Furthermore, differences in the handling of covariates, thresholds used to define hypertension, and modelling strategies may also have contributed. These methodological differences may explain why similar analyses of UKB data yielded contrasting results. It is also important to recognize that the UKB cohort is predominantly of White European ancestry (>97%), which limits the generalizability of these findings to other ancestries. Hypertension prevalence, BP trajectories, myocardial remodelling patterns and fibrosis burden vary across populations. Validation in more diverse cohorts, including populations with higher prevalence of hypertension-related organ damage, will be essential to establish the broader applicability of these CMR-derived phenotypes.

There have also been smaller clinical studies characterizing the hypertensive phenotype using CMR which support our findings. Rodrigues *et al*.^3^ assessed myocardial and aortic function in 88 hypertensive patients compared with 29 age and sex matched controls. They demonstrated that eccentric LVH had the most advanced impairment in LV and RV function, consistent with our results. They also showed that patients with concentric LVH had highest T1 values compared with the other hypertension-mediated LVH phenotypes. Similarly, in their single centre study of 46 hypertensive patients, Treibel *et al*.^4^ showed that T1 mapping-revealed increased diffuse myocardial fibrosis only occurred in hypertensives with LVH. This clinical cohort was not further differentiated into the LVH phenotypes.

More recently Canciello *et al*.^23^ evaluated sex-related differences in hypertension-mediated LVH using echocardiography from a prospective registry of 6427 Italian hypertensive patients (43% female) without prevalent cardiovascular disease. At baseline, females had a lower prevalence of normal LV geometry (50% vs. 72%) than males and higher prevalence of eccentric LVH (40% vs. 18%). At long-term follow-up (mean 6.1 years), normal LV remained less frequent in females than males (43% vs. 67%) with differences in eccentric (41% vs. 21%) and concentric LVH (11% vs. 5%) persisting. Our study also showed females had a lower prevalence of normal LV than males (45% vs. 55%); however, a lower prevalence of eccentric LVH (42% vs. 58%) at baseline. We also found a higher prevalence of concentric LVH in females (59% vs. 41%). These differences may in part be due to clinical characteristics as the Campania Salute network registry is a clinical cohort and imaging analysis was performed using echocardiography. Furthermore, the Italian cohort was younger (mean age 53 years) compared with UKB (mean age 67 years) which may in part also explain the differences.

### Associations with clinical outcomes

This is the first study to explore the prognostic implications of CMR-derived LV geometry in hypertensive subjects in UKB. Notably, hypertensives with eccentric LVH had a nine-fold increase in hazard rate of heart failure compared with those with normal LV geometry. There were no significant associations with LV remodelling phenotype and clinical outcomes. The cumulative survival probability was also worse in participants with eccentric LVH.

There have been other smaller studies comparing prognosis between concentric and eccentric LVH in other longitudinal CMR cohorts. Ha *et al*. explored associations with long-term mortality outcomes of eccentric and concentric LVH in 4979 subjects of the Multi-Ethnic Study of Atherosclerosis (MESA). The authors found that the primary end point of all-cause mortality (*n* = 1,137, 22.8%) occurred in 47.4%, and 56.6% of participants with concentric LVH, and eccentric LVH, respectively (*P* < 0.001).^24^ Due to the relatively short follow-up period in UKB from the imaging visit, we did not explore associations with mortality in this study. Future work will extend this approach by validating our findings as event numbers increase in UKB and by leveraging the repeat imaging to test surrogate imaging endpoints for risk stratification. This will be particularly informative given the potential for residual confounding from unmeasured factors such as hypertension duration, treatment class, kidney function and lifestyle characteristics, and considering our sensitivity analysis in which some associations reached statistical significance in the absence of BP adjustment. It is also important to recognize that the UKB cohort is predominantly of White European ancestry (>97%), which limits the generalizability of these findings to other ancestries. Hypertension prevalence, BP trajectories, myocardial remodelling patterns and fibrosis burden vary across populations. Validation in more diverse cohorts, including populations with higher prevalence of hypertension-related organ damage, will be essential to establish the broader applicability of these CMR-derived phenotypes.

Garg *et al.*^25^ conducted a study to determine cardiovascular outcomes (heart failure and death) in the community population of the Dallas Heart Study using a 4-tiered CMR classification of LVH. Eccentric LVH was further subdivided into ‘indeterminate hypertrophy’ and ‘dilated hypertrophy’ and concentric LVH into ‘thick hypertrophy’ and ‘both thick and dilated hypertrophy,’ based on the presence of increased LV end-diastolic volume.^26^ Over a median follow-up period of 9 years, there were 35 heart failure events and 47 deaths, in a cohort of 2458 participants with 31% prevalence of hypertension. Compared with participants without LVH, those with eccentric dilated (HR 7.3; 95% CI: 2.8–8.8), thick (HR: 2.4; 95% CI: 1.4–4.0), and both thick and dilated (HR: 5.8; 95% CI: 1.7–19.5) hypertrophy remained at increased risk for heart failure or cardiovascular death, compared with the group with indeterminate hypertrophy (HR: 0.9; 95% CI: 0.4–2.2). This study highlights further imaging characterization of the extremes of LVH, to add further granularity and identify differences between eccentric and concentric LVH. This approach may be for future work for further exploration of the participants exhibiting these phenotypes in the UKB.

### Study limitations

An important limitation is that despite using an established CMR classification of hypertension-mediated LVH phenotypes, a small proportion of participants were uncategorized. These individuals either had raised LVM or raised LVEDV with the other LV parameters being in the normal range, which did not correspond to any of the four LVH categories (*n* = 1099). This group had discordant mass-to-volume characteristics not aligning with any defined phenotype. There is important nuance in distinguishing discrete pathophysiologic entities from variations along a continuous structural and functional spectrum. Our use of categorical LVH phenotypes was informed by existing CMR-based classification schemes that reflect traditional mass-to-volume geometric models. While this framework offers interpretability and clinical familiarity, the boundaries between phenotypes are inherently influenced by the thresholds applied. Our findings do not replicate a clear demographic clustering (e.g. LV remodelling predominating in older women), and we note substantial clinical overlap between groups. This likely reflects both the arbitrariness of cut-offs and the influence of shared pathophysiologic drivers (e.g. age, BP burden, treatment response) that act along a continuum. It is therefore important to note that there are other CMR LVH categorization systems but these have not been used specifically for hypertensive individuals.^2,26^ This raises the question whether CMR metrics such as T1 values and LA volumes should be considered when categorizing hypertensives based on imaging parameters.

An additional methodological consideration relates to the handling of BP in participants receiving anti-hypertensive treatment. We applied a treatment adjustment to systolic and diastolic BP, consistent with established epidemiological practice for approximating underlying BP exposure. However, this adjusted value does not reflect the actual haemodynamic load at the time of CMR acquisition. As ventricular structure and function are load dependent, use of treatment-adjusted rather than measured contemporaneous BP may lead to misrepresentation of afterload in models evaluating CMR-derived parameters. Although this approach was intended to reduce misclassification of lifetime hypertensive burden, we acknowledge that it may partly influence effect estimates and should be interpreted with caution. BP ascertainment was based on measurements obtained at a single imaging visit, supplemented by self-reported hypertension, physician diagnosis and anti-hypertensive medication use. While this approach aims at reducing the effect of white-coat or masked hypertension, some misclassifications may persist.

It is also important to highlight that event rates in this cohort were low and the eccentric and concentric LVH groups were relatively small, reflecting the generally healthy nature of the UKB population. As a result, some analyses, particularly those involving concentric LVH and MACE may have risk of type II error. Non-significant results in these strata should therefore be interpreted cautiously, as the wide confidence intervals may have clinically meaningful effects. Moreover, the lack of ethnic diversity in the UKB cohort (>97% White European), which restricts the generalizability of our findings. Therefore, validation in more ethnically diverse populations is needed to determine whether these CMR-defined LVH phenotypes and their prognostic implications extend beyond European ancestry. Finally, as our study explores associations between hypertension-mediated LVH phenotypes, conclusions based on causation cannot be drawn.

### Clinical perspectives

The correlation between BP measurements and LV mass is modest, particularly in individuals established on anti-hypertensive therapy.^27^ This highlights the challenges of recommending optimal BP targets for hypertension-mediated LVH. In hypertension, the myocardium hypertrophies in response to elevated BP, initially as an adaptive process with cardiomyocyte growth and compensatory hypertrophy. If the stimulus is maintained, the response becomes maladaptive with cardiomyocyte death and fibrosis which results in progressive LV dysfunction leading ultimately to heart failure.^28^ Non-invasive imaging techniques such as T1 mapping allow for quantifying myocardial fibrosis. These imaging markers may be used as surrogate endpoints to risk stratify hypertensive individuals with LVH. Furthermore, as LVH can regress with appropriate anti-hypertensive treatment our findings may help guide future therapies specific to each LVH phenotype. Upregulation of the renin-angiotensin-aldosterone system (RAAS) is a known determinant of LVH and myocardial fibrosis.^29^ In addition, concentrations of cardiac natriuretic peptides are elevated in LVH and represent a potential therapeutic target.^30^ REVERSE-LVH is an ongoing clinical trial, designed to compare the effects of 52 weeks of treatment with sacubitril/valsartan (combination neprilysin and angiotensin receptor inhibitor) with valsartan on the primary endpoint of change in interstitial volume measured by CMR in patients with hypertension and LVH.^31^ These clinical trials guided by imaging-derived endpoints place greater emphasis on identifying at risk hypertensive individuals with LVH, and targeting treatment irrespective of BP.

## Conclusion

In this large population study, we have shown distinct differences in CMR structural and function characteristics among hypertension-mediated LVH phenotypes. We have also shown that among hypertensives, eccentric and concentric LVH have worse cardiovascular outcomes. The imaging markers in this study may be used as surrogate endpoints to risk stratify hypertensives with LVH.

## Supplementary Material

qyaf168_Supplementary_Data

## Data Availability

This research was conducted using the UK Biobank resource under access application 2964. UK Biobank will make the data available to all bona fide researchers for all types of health-related research that is in the public interest, without preferential or exclusive access for any persons. All researchers will be subject to the same application process and approval criteria as specified by UK Biobank. For more details on the access procedure, see the UK Biobank website: http://www.ukbiobank.ac.uk/register-apply. This work uses data provided by patients and collected by the NHS as part of their care and support. This research used data assets made available by National Safe Haven as part of the Data and Connectivity National Core Study, led by Health Data Research UK in partnership with the Office for National Statistics and funded by UK Research and Innovation (research which commenced between 1st October 2020–31st March 2021 grant ref MC_PC_20029; 1st April 2021 −30th September 2022 grant ref MC_PC_20058).

## Lead author biography

**Hafiz Naderi** is an Academic Clinical Lecturer in Cardiology at Queen Mary University of London and Barts Heart Centre, funded by the National Institute for Health and Care Research. His clinical and research interests focus on hypertension, cardiovascular imaging, and the application of artificial intelligence to improve early detection and risk stratification. In 2024, he was awarded a PhD in Cardiovascular Medicine, funded by the British Heart Foundation, on machine learning approaches to predict hypertension-mediated subclinical left ventricular hypertrophy. His work has received several early-career awards and he contributes to national and international hypertension research and education through roles within the British and Irish Hypertension Society and the European Society of Hypertension.
