# Supplementary material for: Cardiac magnetic resonance characteristics and prognostic associations of hypertension-mediated left ventricular hypertrophy
Source: Eur Heart J Imaging Methods Pract. 2026 Jan 13;4(1):qyaf168. doi: 10.1093/ehjimp/qyaf168 (PMC12822606; doi:10.1093/ehjimp/qyaf168)
Supplement: qyaf168_Supplementary_Data [file qyaf168_supplementary_data.docx]

**Supplementary material**

**Supplementary Table 1.** UK Biobank data fields used to identify hypertensive participants.

| **Phenotype** | **Data fields** | **Field names** | **Data code definitions** |
| --- | --- | --- | --- |
| Hypertension | 20002 | Non-cancer illness code, self-reported | Hypertension, Essential hypertension |
| Hypertension | 6150 | Vascular/heart problems diagnosed by doctor | High blood pressure |
| Hypertension | 6177 | Medication for cholesterol, blood pressure or diabetes | Blood pressure medication |
| Hypertension | 6153 | Medication for cholesterol, blood pressure, diabetes or take exogenous hormones | Blood pressure medication |
| Hypertension | 4080 | Systolic blood pressure, automated reading | ≥130 mmHg |
| Hypertension | 4079 | Diastolic blood pressure, automated reading | ≥85 mmHg |
| Hypertension | 93 | Systolic blood pressure, manual reading | ≥130 mmHg |
| Hypertension | 94 | Diastolic blood pressure, manual reading | ≥85 mmHg |
| Aortic stenosis | 20002 | Non-cancer illness code, self-reported | Aortic stenosis |
| Hypertrophic cardiomyopathy | 20002 | Non-cancer illness code, self-reported | Hypertrophic cardiomyopathy |

**Supplementary Table 2.** Codes used to define clinical outcomes using ICD-9, ICD-10and OPCS4 codes.

| **Myocardial Infarction** | |
| --- | --- |
| **ICD10 codes** | **Definition** |
| I21 | Acute myocardial infarction |
| I21.0 | Acute transmural myocardial infarction of anterior wall |
| I21.1 | Acute transmural myocardial infarction of inferior wall |
| I21.2 | Acute transmural myocardial infarction of other sites |
| I21.3 | Acute transmural myocardial infarction of unspecified site |
| I21.4 | Acute subendocardial myocardial infarction |
| I21.9 | Acute myocardial infarction, unspecified |
| I22 | Subsequent myocardial infarction |
| I22.0 | Subsequent myocardial infarction of anterior wall |
| I22.1 | Subsequent myocardial infarction of inferior wall |
| I22.8 | Subsequent myocardial infarction of other sites |
| I22.9 | Subsequent myocardial infarction of unspecified site |
| I23 | Certain current complications following acute myocardial infarction |
| I23.0 | Haemopericardium as current complication following acute myocardial infarction |
| I23.1 | Atrial septal defect as current complication following acute myocardial infarction |
| I23.2 | Ventricular septal defect as current complication following acute myocardial infarction |
| I23.3 | Rupture of cardiac wall without haemoprericardium as current complication following acute myocardial infarction |
| I23.5 | Rupture of papillary muscle as current complication following acute myocardial infarction |
| I23.6 | Thrombosis of atrium, auricular appendage and ventricle as current complications following acute myocardial infarction |
| I23.8 | Other current complications following acute myocardial infarction |
| I24 | Other acute ischaemic heart disease |
| I24.0 | Coronary thrombosis not resulting in myocardial infarction |
| I24.1 | Dressler’s syndrome |
| I24.8 | Other forms of acute ischaemic heart disease |
| I24.9 | Acute ischaemic heart disease, unspecified |
| I25 | Chronic ischaemic heart disease |
| I25.0 | Atherosclerotic cardiovascular disease |
| I25.1 | Atherosclerotic heart disease |
| I25.2 | Old myocardial infarction |
| I25.3 | Aneurysm of heart |
| I25.4 | Coronary artery aneurysm |
| I25.5 | Ischaemic cardiomyopathy |
| I25.6 | Silent myocardial ischaemia |
| I25.8 | Other forms of chronic ischaemic heart disease |
| I25.9 | Chronic ischaemic heart disease, unspecified |
| **ICD9 codes** | **Definition** |
| 4109 | Acute myocardial infarction |
| **OPCS4** | **Definition** |
| K40 | Saphenous vein graft replacement of coronary artery |
| K40.1 | Saphenous vein graft replacement of one coronary artery |
| K40.2 | Saphenous vein graft replacement of two coronary arteries |
| K40.3 | Saphenous vein graft replacement of three coronary arteries |
| K40.4 | Saphenous vein graft replacement of four or more coronary arteries |
| K40.8 | Other specified saphenous vein graft replacement of coronary artery |
| K40.9 | Unspecified saphenous vein graft replacement of coronary artery |
| K41 | Other autograft replacement of coronary artery |
| K41.1 | Autograft replacement of one coronary artery NEC |
| K41.2 | Autograft replacement of two coronary arteries NEC |
| K41.3 | Autograft replacement of three coronary arteries NEC |
| K41.4 | Autograft replacement of four or more coronary arteries NEC |
| K41.8 | Other specified other autograft replacement of coronary artery |
| K41.9 | Unspecified other autograft replacement of coronary artery |
| K42 | Allograft replacement of coronary artery |
| K42.1 | Allograft replacement of one coronary artery |
| K42.2 | Allograft replacement of two coronary arteries |
| K42.3 | Allograft replacement of three coronary arteries |
| K42.4 | Allograft replacement of four or more coronary arteries |
| K42.8 | Other specified other allograft replacement of coronary artery |
| K42.9 | Unspecified other allograft replacement of coronary artery |
| K44 | Other replacement of coronary artery |
| K44.1 | Replacement of coronary arteries using multiple methods |
| K44.2 | Revision of replacement of coronary artery |
| K44.8 | Other specified other replacement of coronary artery |
| K44.9 | Unspecified other replacement of coronary artery |
| K45 | Connection of thoracic artery to coronary artery |
| K45.1 | Double anastomosis of mammary arteries to coronary arteries |
| K45.2 | Double anastomosis of thoracic arteries to coronary arteries NEC |
| K45.3 | Anastomosis of mammary artery to left anterior descending coronary artery |
| K45.4 | Anastomosis of mammary artery to coronary artery NEC |
| K45.5 | Anastomosis of thoracic artery to coronary artery NEC |
| K45.6 | Revision of connection of thoracic artery to coronary artery |
| K45.8 | Other specified connection of thoracic artery to coronary artery |
| K45.9 | Unspecified connection of thoracic artery to coronary aartery |
| K49 | Transluminal balloon angioplasty of coronary artery |
| K49.1 | Percutaneous transluminal balloon angioplasty of one coronary artery |
| K49.2 | Percutaneous transluminal balloon angioplasty of multiple coronary arteries |
| K49.3 | Percutaneous transluminal balloon angioplasty of bypass graft of coronary artery |
| K49.4 | Percutaneous transluminal cutting balloon angioplasty of coronary artery |
| K49.8 | Other specified transluminal balloon angioplasty of coronary artery |
| K49.9 | Unspecified transluminal balloon angioplasty of coronary artery |
| K50 | Other therapeutic transluminal operations on coronary artery |
| K50.1 | Percutaneous transluminal laser coronary angioplasty |
| K50.2 | Percutaneous transluminal coronary thrombolysis using streptokinase |
| K50.3 | Percutaneous transluminal injection of therapeutic substance into coronary artery NEC |
| K50.4 | Percutaneous transluminal atherectomy of coronary artery |
| K50.8 | Other specified other therapeutic transluminal; operations on coronary artery |
| K50.9 | Unspecified other therapeutic transluminal operations on coronary artery |
| K75 | Percutaneous transluminal balloon angioplasty and insertion of stent into coronary artery |
| K75.1 | Percutaneous transluminal balloon angioplasty and insertion of 1-2 drug-eluting stents into coronary artery |
| K75.2 | Percutaneous transluminal balloon angioplasty and insertion of 3 or more drug-eluting stents into coronary artery |
| K75.3 | Percutaneous transluminal balloon angioplasty and insertion of 1-2 stents into coronary artery |
| K75.4 | Percutaneous transluminal balloon angioplasty and insertion of 3 or more stents into coronary artery NEC |
| K75.8 | Other specified percutaneous transluminal balloon angioplasty and insertion of stent into coronary artery |
| K75.9 | Unspecified percutaneous transluminal balloon angioplasty and insertion of stent into coronary artery |
| **Heart Failure** | |
| **ICD10 codes** | **Definition** |
| I11.0 | Hypertensive heart disease with (congestive) heart failure |
| I13.0 | Hypertensive heart and renal disease with (congestive) heart failure |
| I13.2 | Hypertensive heart and renal disease with both (congestive) heart failure and renal failure |
| I25.5 | Ischaemic cardiomyopathy |
| I50 | Heart failure |
| I50.0 | Congestive heart failure |
| I50.1 | Left ventricular failure |
| I50.9 | Heart failure, unspecified |
| J81 | Pulmonary oedema |
| K76.1 | Chronic passive congestion of liver |
| **ICD9 codes** | **Definition** |
| 4280 | Congestive heart failure |
| 4281 | Left heart failure |
| 4289 | Heart failure, unspecified |
| **OPCS4 codes** | **Definition** |
| K59.6 | Implantation of cardioverter defibrillator using three electrode leads |
| K61.7 | Implantation of biventricular cardiac pacemaker system |
| K60.7 | Implantation of intravenous biventricular cardiac pacemaker system |
| **Stroke** | |
| **ICD10 codes** | **Definition** |
| I60 | Subarachnoid haemorrhage |
| I60.0 | Subarachnoid haemorrhage from carotid siphon bifurcation |
| I60.1 | Subarachnoid haemorrhage from middle cerebral artery |
| I60.2 | Subarachnoid haemorrhage from anterior communicating artery |
| I60.3 | Subarachnoid haemorrhage from posterior communicating artery |
| I60.4 | Subarachnoid haemorrhage from basilar artery |
| I60.5 | Subarachnoid haemorrhage from vertebral artery |
| I60.6 | Subarachnoid haemorrhage from other intracranial arteries |
| I60.7 | Subarachnoid haemorrhage from intracranial artery, unspecified |
| I60.8 | Other subarachnoid haemorrhage |
| I60.9 | Subarachnoid haemorrhage, unspecified |
| I61 | Intracerebral haemorrhage |
| I61.0 | Intracerebral haemorrhage in hemisphere subcortical |
| I61.1 | Intracerebral haemorrhage in hemisphere, cortical |
| I61.2 | Intracerebral haemorrhage in hemisphere, unspecified |
| I61.3 | Intracerebral haemorrhage in brain stem |
| I61.4 | Intracerebral haemorrhage in cerebellum |
| I61.5 | Intracerebral haemorrhage, intraventricular |
| I61.6 | Intracerebral haemorrhage, multiple localised |
| I61.8 | Other intracerebral haemorrhage |
| I61.9 | Intracerebral haemorrhage, unspecified |
| I62 | Other nontraumatic intracranial haemorrhage |
| I62.0 | Subdural haemorrhage (acute) (nontraumatic) |
| I62.1 | Nontraumatic extradural haemorrhage |
| I62.9 | Intracranial haemorrhage (nontraumatic), unspecified |
| I63 | Cerebral infarction |
| I63.0 | Cerebral infarction due to thrombosis of precerebral arteries |
| I63.1 | Cerebral infarction due to embolism of precerebral arteries |
| I63.2 | Cerebral infarction due to unspecified occlusion or stenosis of precerebral arteries |
| I63.3 | Cerebral infarction due to thrombosis of cerebral arteries |
| I63.4 | Cerebral infarction due to embolism of cerebral arteries |
| I63.5 | Cerebral infarction due to unspecified occlusion or stenosis of cerebral arteries |
| I63.6 | Cerebral infarction due to cerebral venous thrombosis, nonpyogenic |
| I63.8 | Other cerebral infarction |
| I63.9 | Cerebral infarction, unspecified |
| I64 | Stroke, not specified as haemorrhage or infarction |
| I65 | Occlusion and stenosis of precerebral arteries, not resulting in cerebral infarction |
| I65.0 | Occlusion and stenosis of vertebral artery |
| I65.1 | Occlusion and stenosis of basilar artery |
| I65.2 | Occlusion and stenosis of carotid artery |
| I65.3 | Occlusion and stenosis of multiple and bilateral precerebral arteries |
| I65.8 | Occlusion and stenosis of other precerebral artery |
| I65.9 | Occlusion and stenosis of unspecified precerebral artery |
| I66 | Occlusion and stenosis of cerebral arteries, not resulting in cerebral infarction |
| I66.0 | Occlusion and stenosis of middle cerebral artery |
| I66.1 | Occlusion and stenosis of anterior cerebral artery |
| I66.2 | Occlusion and stenosis of posterior cerebral artery |
| I66.3 | Occlusion and stenosis of cerebellar arteries |
| I66.4 | Occlusion and stenosis of multiple and bilateral cerebral arteries |
| I66.8 | Occlusion and stenosis of other cerebral artery |
| I66.9 | Occlusion and stenosis of unspecified cerebral artery |
| I67.0 | Dissection of cerebral arteries, nonruptured |
| I67.8 | Other specified cerebrovascular diseases |
| I67.9 | Cerebrovascular disease, unspecified |
| I69 | Sequelae of cerebrovascular disease |
| **ICD9 codes** | **Definition** |
| 4309 | Subarachnoid haemorrhage |
| 4319 | Intracerebral haemorrhage |
| 4320 | Nontraumatic extradural haemorrhage |
| 4321 | Subdural haemorrhage |
| 4331 | Occlusion and stenosis of carotid artery |
| 4339 | Occlusion and stenosis of precerebral arteries, unspecified |
| 4349 | Occlusion of cerebral arteries, unspecified |
| 4369 | Acute but ill-defined cerebrovascular disease |
| 4371 | Other generalised ischaemic cerebrovascular disease |
| **OPCS4 codes** | **Definition** |
| L35.4 | Percutaneous transluminal embolectomy of cerebral artery |
| **Ventricular Arrythmias** | |
| **ICD10 codes** | **Definition** |
| I47.2 | Ventricular tachycardia |
| I49.0 | Ventricular fibrillation and flutter |
| I46.0 | Cardiac arrest with successful resuscitation |
| I46.1 | Sudden cardiac death, so described |
| I46.9 | Cardiac arrest, unspecified |
| I47.0 | Re-entry ventricular arrythmia |
| **ICD9 codes** | **Definition** |
| 4270 | Paroxysmal ventricular tachycardia |
| 4271 | Paroxysmal ventricular tachycardia |
| 4272 | Paroxysmal tachycardia, unspecified |
| 4274 | Ventricular fibrillation and flutter |
| **OPCS4 codes** | **Definition** |
| K59 | Cardioverter defibrillator introduced through vein |
| K59.1 | Implantation of cardioverter defibrillator using one electrode lead |
| K59.2 | Implantation of cardioverter defibrillator using two electrode leads |
| K59.3 | Resitting of leads of cardioverter defibrillator |
| K59.4 | Renewal of cardioverter defibrillator |
| K59.6 | Implantation of cardioverter defibrillator using three electrode leads |
| K59.8 | Other specified cardioverter defibrillator introduced through the vein |
| K59.9 | Unspecified cardioverter defibrillator introduced through the vein |
| K72 | Other cardioverter defibrillator |
| K72.1 | Implantation of subcutaneous cardioverter defibrillator |
| K72.3 | Renewal of subcutaneous cardioverter defibrillator |

**Supplementary Table 3.** Post hoc pairwise comparisons of key CMR parameters across hypertension-mediated LVH phenotypes.

| **CMR parameters** | **Comparison** | **Mean difference** | **95% CI lower** | **95% CI upper** | **p-value** |
| --- | --- | --- | --- | --- | --- |
| GLS | LV remodelling-Normal LV | 1.4 | 1.2 | 1.6 | <0.001 |
| GLS | Eccentric LVH-Normal LV | 0.9 | 0.6 | 1.3 | <0.001 |
| GLS | Concentric LVH-Normal LV | 1.9 | 1.4 | 2.4 | <0.001 |
| GLS | Eccentric LVH-LV remodelling | -0.5 | -0.9 | -0.1 | 0.012 |
| GLS | Concentric LVH-LV remodelling | 0.4 | -0.1 | 1.0 | 0.156 |
| GLS | Concentric LVH-Eccentric LVH | 0.9 | 0.3 | 1.6 | <0.001 |
| T1 mapping | LV remodelling-Normal LV | 3.6 | 0.8 | 6.4 | 0.006 |
| T1 mapping | Eccentric LVH-Normal LV | 4.5 | -0.6 | 9.6 | 0.103 |
| T1 mapping | Concentric LVH-Normal LV | 18.8 | 11.0 | 26.7 | <0.001 |
| T1 mapping | Eccentric LVH-LV remodelling | 0.9 | -4.8 | 6.7 | 0.975 |
| T1 mapping | Concentric LVH-LV remodelling | 15.3 | 6.9 | 23.6 | <0.001 |
| T1 mapping | Concentric LVH-Eccentric LVH | 14.3 | 5.0 | 23.7 | <0.001 |
| LVEF | LV remodelling-Normal LV | 1.4 | 0.9 | 1.9 | <0.001 |
| LVEF | Eccentric LVH-Normal LV | -4.1 | -5.2 | -3.1 | <0.001 |
| LVEF | Concentric LVH-Normal LV | 0.6 | -0.8 | 2.0 | 0.706 |
| LVEF | Eccentric LVH-LV remodelling | -5.5 | -6.7 | -4.4 | <0.001 |
| LVEF | Concentric LVH-LV remodelling | -0.8 | -2.3 | 0.7 | 0.508 |
| LVEF | Concentric LVH-Eccentric LVH | 4.7 | 3.0 | 6.5 | <0.001 |
| LVSV | LV remodelling-Normal LV | -18.2 | -19.8 | -16.7 | <0.001 |
| LVSV | Eccentric LVH-Normal LV | 17.9 | 14.7 | 21.1 | <0.001 |
| LVSV | Concentric LVH-Normal LV | 1.7 | -2.5 | 5.9 | 0.720 |
| LVSV | Eccentric LVH-LV remodelling | 36.1 | 32.6 | 39.6 | <0.001 |
| LVSV | Concentric LVH-LV remodelling | 20.0 | 15.5 | 24.4 | <0.001 |
| LVSV | Concentric LVH-Eccentric LVH | -16.1 | -21.4 | -10.9 | <0.001 |
| RVEF | LV remodelling-Normal LV | -0.7 | -1.2 | -0.2 | 0.004 |
| RVEF | Eccentric LVH-Normal LV | -3.1 | -4.1 | -2.1 | <0.001 |
| RVEF | Concentric LVH-Normal LV | 0.5 | -0.9 | 1.9 | 0.832 |
| RVEF | Eccentric LVH-LV remodelling | -2.4 | -3.5 | -1.3 | <0.001 |
| RVEF | Concentric LVH-LV remodelling | 1.2 | -0.4 | 2.7 | 0.200 |
| RVEF | Concentric LVH-Eccentric LVH | 3.6 | 1.9 | 5.3 | <0.001 |
| LA size | LV remodelling-Normal LV | -5.2 | -6.3 | -4.1 | <0.001 |
| LA size | Eccentric LVH-Normal LV | 10.5 | 8.4 | 12.6 | <0.001 |
| LA size | Concentric LVH-Normal LV | 4.1 | 1.2 | 7.0 | 0.002 |
| LA size | Eccentric LVH-LV remodelling | 15.7 | 13.4 | 18.0 | <0.001 |
| LA size | Concentric LVH-LV remodelling | 9.3 | 6.2 | 12.4 | <0.001 |
| LA size | Concentric LVH-Eccentric LVH | -6.4 | -10.0 | -2.8 | <0.001 |
| RA size | LV remodelling-Normal LV | -8.1 | -9.4 | -6.8 | <0.001 |
| RA size | Eccentric LVH-Normal LV | 8.6 | 6.0 | 11.2 | <0.001 |
| RA size | Concentric LVH-Normal LV | -3.1 | -6.7 | 0.4 | 0.106 |
| RA size | Eccentric LVH-LV remodelling | 16.7 | 13.9 | 19.5 | <0.001 |
| RA size | Concentric LVH-LV remodelling | 5.0 | 1.2 | 8.7 | 0.004 |
| RA size | Concentric LVH-Eccentric LVH | -11.7 | -16.1 | -7.4 | <0.001 |
| LV wall thickness | LV remodelling-Normal LV | 1.4 | 1.3 | 1.6 | <0.001 |
| LV wall thickness | Eccentric LVH-Normal LV | 1.3 | 1.1 | 1.6 | <0.001 |
| LV wall thickness | Concentric LVH-Normal LV | 1.9 | 1.5 | 2.4 | <0.001 |
| LV wall thickness | Eccentric LVH-LV remodelling | -0.1 | -0.4 | 0.1 | 0.650 |
| LV wall thickness | Concentric LVH-LV remodelling | 0.5 | 0.1 | 0.9 | 0.020 |
| LV wall thickness | Concentric LVH-Eccentric LVH | 0.6 | 0.1 | 1.1 | 0.006 |

*CI: Confidence interval; GLS: Global longitudinal strain; LA: Left atrial; LV: Left ventricle; LVSV: Left ventricular stroke volume; LVEF: Left ventricular ejection fraction; LV LVH left ventricular hypertrophy; RA: right atrial; RVEF: Right ventricular ejection fraction.*

**Supplementary Table 4.** Associations of hypertension-mediated LVH phenotypes and clinical outcomes.

|  | **MACE** | | **Heart Failure** | |
| --- | --- | --- | --- | --- |
|  | **HR (95% CI)** | **P-value** | **HR (95% CI)** | **P-value** |
| **LV remodelling** | 1.2 (0.9-1.6) | 0.1 | 1.5 (0.9-2.4) | 0.1 |
| **Eccentric LVH** | 2.5 (1.7-3.8) | <0.001 | 9.0 (5.7-14.2) | <0.001 |
| **Concentric LVH** | 1.8 (1.0-3.3) | 0.07 | 4.1 (1.8-9.3) | <0.001 |

*Results are from Cox proportional hazards regression models with outcomes of interest set as the model outcome (response variable). Hypertension LVH phenotype is the exposure of interest with normal LV as the reference group. There was adjustment for age, sex and BMI, systolic BP, diabetes, high cholesterol and smoking status.*

*HR: Hazard ratio, CI: Confidence interval.*

**Supplementary Table 5.** Associations of hypertension-mediated LVH phenotypes and clinical outcomes without BP adjustment of participants on anti-hypertensive therapy.

|  | **MACE** | | **Heart Failure** | |
| --- | --- | --- | --- | --- |
|  | **HR (95% CI)** | **P-value** | **HR (95% CI)** | **P-value** |
| **LV remodelling** | 1.3 (1.0-1.8) | 0.04 | 1.6 (0.9-2.4) | 0.05 |
| **Eccentric LVH** | 2.3 (1.4-3.5) | <0.001 | 10.6 (6.7-16.9) | <0.001 |
| **Concentric LVH** | 1.9 (1.0-3.6) | 0.04 | 4.0 (1.7-9.9) | <0.001 |

*Results are from Cox proportional hazards regression models with outcomes of interest set as the model outcome (response variable). Hypertension LV phenotype is the exposure of interest with normal LV as the reference group. There was adjustment for age, sex and BMI, systolic BP, diabetes, high cholesterol and smoking status.*

*HR: Hazard ratio, CI: Confidence interval.*

**Supplementary Figure 1.** Correlation matrix heatmap of CMR measures.


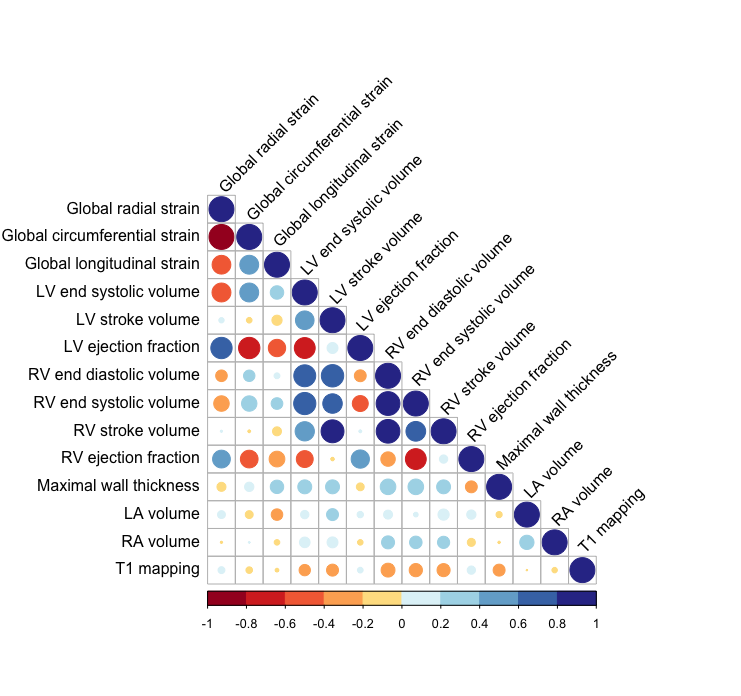


*Shades of blue indicate a positive correlation, and shades of red represent a negative correlation. Size and shade of each circle indicate the strength of correlation*
